# Addressing misclassification bias in vaccine effectiveness studies with an application to Covid-19

**DOI:** 10.1186/s12874-023-01853-4

**Published:** 2023-02-27

**Authors:** Paolo Eusebi, Niko Speybroeck, Sonja Hartnack, Jacob Stærk-Østergaard, Matthew J. Denwood, Polychronis Kostoulas

**Affiliations:** 1 Department of Medicine and Surgery, University of Perugia, Perugia, Italy; 2 Modus Outcomes, a division of THREAD, Lyon, France; 3 Institute of Health and Society, Université catholique de Louvain, Brussels, Belgium; 4 Section of Epidemiology, Vetsuisse Faculty, University of Zurich, Zurich, Switzerland; 5 Department of Veterinary and Animal Sciences, University of Copenhagen, Copenhagen, Denmark; 6 Faculty of Public Health, University of Thessaly, Thessaly, Greece

**Keywords:** RT-PCR, Test-negative design, Sensitivity, Specificity, Covid-19

## Abstract

Safe and effective vaccines are crucial for the control of Covid-19 and to protect individuals at higher risk of severe disease. The test-negative design is a popular option for evaluating the effectiveness of Covid-19 vaccines. However, the findings could be biased by several factors, including imperfect sensitivity and/or specificity of the test used for diagnosing the SARS-Cov-2 infection. We propose a simple Bayesian modeling approach for estimating vaccine effectiveness that is robust even when the diagnostic test is imperfect. We use simulation studies to demonstrate the robustness of our method to misclassification bias and illustrate the utility of our approach using real-world examples.

**Supplementary Information:**

The online version contains supplementary material available at 10.1186/s12874-023-01853-4.

## Background

There is mounting evidence that the authorized vaccines for severe acute respiratory syndrome coronavirus 2 (SARS-CoV-2) are safe and effective. However, it is crucial to continuously assess the effectiveness of vaccination programs using valid statistical methods. Several study designs can be utilized for evaluating vaccine effectiveness (VE). One popular option is the test-negative design (TND), which has commonly been used for published post-licensure vaccine studies [1–3].

The TND involves comparing individuals who test positive and those who test negative for SARS-CoV-2 in both vaccinated and unvaccinated subgroups. The study participants typically have similar signs and symptoms and are usually selected from the same population (e.g., persons who seek and have access to medical care). Due to their suitability for use with large electronic health databases and their relatively low logistical burden, TNDs have been widely implemented for monitoring the effectiveness of Covid-19 vaccines [4]. In these studies, the reverse transcriptase-polymerase chain reaction (RT-PCR) test is routinely used to classify the individual patients as being negative or positive for SARS-CoV-2.

Despite its widespread use, the TND has some limitations, including the risk of bias arising from misclassification of cases and negative controls due to the imperfect nature of the diagnostic test [2, 4]. Although RT-PCR has a high specificity for SARS-CoV-2, the sensitivity can vary with several factors [2]. One modeling study showed that the sensitivity ranged from 0.62 (95% probability interval (PrI)=0.35-0.82) on the day of symptom onset to 0.80 (95% PrI=0.70-0.88) three days after symptom onset [5]. In a pooled analysis of studies assessing the accuracy of RT-PCR and serological tests for Covid-19 diagnosis, Kostoulas and colleagues [6] estimated a sensitivity of 0.68 (95% PrI=0.63-0.73) and a specificity of 0.99 (95% PrI=0.98-1.00) for RT-PCR. The real-world performance of RT-PCR and antigen testing has also been estimated in a further study using data from the Danish national registries. Here, the estimated specificity of RT-PCR was greater than 0.997, while the sensitivity was estimated to be 0.957 (95% PrI=0.928-984) [7]. This study found no evidence that RT-PCR sensitivity differed between vaccinated and unvaccinated individuals, although the number of test-positive vaccinated individuals was small.

In this paper, we demonstrate the implementation of simple Bayesian models for addressing potential misclassification bias in TND studies that seek to evaluate the effectiveness of Covid-19 vaccines. Prior information about RT-PCR sensitivity and specificity is incorporated into the model. Although our illustrated example focuses on TND studies for Covid-19 vaccines, our statistical approach is directly applicable to TND studies for other diseases, and can also be generalized to other study designs (e.g., randomized clinical trials, cohort and case-control studies).

The paper is structured as follows. We begin by briefly describing the existing TND studies for VE. We then propose a simple Bayesian modeling framework for addressing misclassification bias. Following this, a simulation exercise is performed to investigate the impact of misclassification bias on TND studies assessing the effectiveness of Covid-19 vaccines, including an evaluation of the ability of our proposed method to provide unbiased estimates. Finally, an illustrative example of the method is presented using data from a TND study performed in Ontario, Canada, to investigate the effectiveness of mRNA Covid-19 vaccines against symptomatic SARS-CoV-2 infection. The VE is estimated using different scenarios for sensitivity and specificity of RT-PCR for this example application.

## Methods

### Studies using a test-negative design to assess vaccine effectiveness

The objective of the TND is to assess the effectiveness of vaccines by comparing the odds of obtaining a positive test from vaccinated patients with the odds of obtaining a positive test from unvaccinated patients. To achieve this, the TND compares cases (subjects with the disease of interest) and controls (people without the disease) drawn from the same source population, which is often a pseudo-population such as patients seeking medical care. This makes the TND a relatively low-cost option, and it reduces the confounding effect of healthcare-seeking behavior. Accordingly, the TND has been successfully implemented as an efficient approach for evaluating the effectiveness of influenza vaccines [8].

If a perfect diagnostic assay is available (i.e., sensitivity and specificity equal to 1), we can assume that the results of the test follow an independent binomial sampling distribution for both vaccinated ($V_{+}$) and unvaccinated ($V_{-}$) subgroups:

1 $$\begin{aligned} y_{V_{+}}\sim & {} Bin(n_{V_{+}}, p_{V_{+}})\end{aligned}$$

2 $$\begin{aligned} y_{V_{-}}\sim & {} Bin(n_{V_{-}}, p_{V_{-}}) \end{aligned}$$

where *y* is the number of the positive tests, *n* is the number of patients, and *p* is the probability of having developed the disease in vaccinated ($V_{+}$) or unvaccinated ($V_{-}$) individuals, respectively.

The Odds Ratio (OR) and the VE are defined as follows:

3 $$\begin{aligned} OR=\frac{p_{V+} /(1-p_{V+})}{p_{V-} /(1-p_{V-})} \end{aligned}$$

4 $$\begin{aligned} VE=(1-OR) \cdot 100 \end{aligned}$$

The OR can be estimated using a two-by-two table or a logistic regression model. The latter also allows for adjustment for potential confounding factors (e.g., age, sex, calendar time, and comorbidities).

### Bayesian modeling methods to address misclassification bias

TND results are vulnerable to misclassification bias, which tends to lead to an underestimation of VE when the diagnostic test used in the study is imperfect (i.e., sensitivity and/or specificity are less than 1) [9].

In the simple case of non-differential misclassification bias, both $Se_{V_{+}}=Se_{V_{-}}=Se$ and $Sp_{V_{+}}=Sp_{V_{-}}=Sp$ hold. We can therefore assume that the test results follow an independent binomial sampling distribution in both vaccinated and unvaccinated individuals:

5 $$\begin{aligned} y_{*V_{+}}\sim & {} Bin(n_{V_{+}}, p_{*V_{+}})\end{aligned}$$

6 $$\begin{aligned} y_{*V_{-}}\sim & {} Bin(n_{V_{-}}, p_{*V_{-}}) \end{aligned}$$

with

7 $$\begin{aligned} p_{*V_{+}}= & {} p_{V_{+}} \cdot Se + (1-p_{V_{+}}) \cdot (1-Sp)\end{aligned}$$

8 $$\begin{aligned} p_{*V_{-}}= & {} p_{V_{-}} \cdot Se + (1-p_{V_{-}}) \cdot (1-Sp) \end{aligned}$$

The model described by equations 5-8 is over-parameterized, with four parameters ($p_{V_{+}}, p_{V_{-}}, Se, Sp$) to be estimated with only two independent pieces of information provided by the data, i.e., the apparent prevalences in the vaccinated ($y_{V_{+}}/n_{V_{+}}$) and unvaccinated ($y_{V_{-}}/n_{V_{-}}$) subgroups. As such, these models are only of practical use within a Bayesian framework of inference, as this allows for prior information on diagnostic test characteristics (sensitivity and specificity) to be used along with the observed data.

In the more complex case of differential misclassification bias, we have both $Se_{V_{+}} \ne Se_{V_{-}}$ and $Sp_{V_{+}} \ne Sp_{V_{-}}$. In this case, the number of parameters increases to six ($p_{V_{+}}, p_{V_{-}}, Se_{V_{+}}, Se_{V_{-}}, Sp_{V_{+}}, Sp_{V_{-}}$) and the equations for the apparent probabilities change as follows:

9 $$\begin{aligned} p_{*V_{+}}= & {} p_{V_{+}} \cdot Se_{V_{+}} + (1-p_{V_{+}}) \cdot (1-Sp_{V_{+}})\end{aligned}$$

10 $$\begin{aligned} p_{*V_{-}}= & {} p_{V_{-}} \cdot Se_{V_{-}} + (1-p_{V_{-}}) \cdot (1-Sp_{V_{-}}) \end{aligned}$$

As for the case with non-differential bias, it is necessary to use a Bayesian framework incorporating prior information for this model to be of practical use. However, some prior knowledge about the sensitivity and specificity of the test may be available, in which case informative priors can be entered into the model and posterior inference obtained for the remaining parameters. There are a number of different ways to select and parameterize priors within a Bayesian framework [10–12].

To enter this prior information into the model, hyper-parameters for Beta distributions $Beta(\alpha , \beta )$ can be obtained from PrI obtained from meta-analytic estimates or from experts’ beliefs. Another option is the use of normal distributions $N(\mu , \sigma ^2)$ for the logit transformation of sensitivity and specificity [13]. In general, the choice of the prior distribution will depend on the available evidence, and the choice of prior must be justified as part of the study [14].

### Software

Bayesian models are fitted using JAGS [15] interfaced to R [16] using the runjags package [17]. The PriorGen package was used for deriving prior distributions [18]. The R code needed to replicate the study is publicly available via a GitHub repository https://github.com/paoloeusebi/tnd-vaccine-effectiveness.

## Simulation study

### Setup

We conducted a simulation study to evaluate the ability of the Bayesian models to address different magnitudes of misclassification bias. For each scenario, we ran 1 000 simulations. In all scenarios we assumed the following: a sample size of 10 000 subjects; a ratio of 7:3 between vaccinated and unvaccinated subjects; a disease prevalence of 20% in unvaccinated subjects.

We assumed that specificity is almost perfect (0.99) or perfect (1.00), and that it does not depend on the vaccination status ($Sp=Sp_{V_{+}}=Sp_{V_{-}}$) i.e. there is no misclassification bias for specificity. In contrast, we allowed sensitivity to vary more substantially across scenarios (from 0.925 to 0.975), with either differential ($Se_{V_{+}} \ne Se_{V_{-}}$) or non-differential ($Se=Se_{V_{+}}=Se_{V_{-}}$) misclassification bias. We simulated data according to 16 scenarios as shown in Table 1.

**Table 1:** The 16 simulation scenarios used to evaluate the ability of the Bayesian models to address different degrees of misclassification bias

Misclassification	*Sp*	$\boldsymbol S{\boldsymbol e}_{{\boldsymbol V}_{\boldsymbol{+}}}$	$\boldsymbol S{\boldsymbol e}_{{\mathbf V}_{\boldsymbol{-}}}$	OR	Scenario #
Non-differential	0.99	0.925	0.925	0.1	1
				0.2	2
		0.975	0.975	0.1	3
				0.2	4
	1.00	0.925	0.925	0.1	5
				0.2	6
		0.975	0.975	0.1	7
				0.2	8
Differential	0.99	0.925	0.975	0.1	9
				0.2	10
		0.975	0.925	0.1	11
				0.2	12
	1.00	0.925	0.975	0.1	13
				0.2	14
		0.975	0.925	0.1	15
				0.2	16

$Se_{V_{-}}$=sensitivity in unvaccinated; $Se_{V_{+}}$=sensitivity in vaccinated; *Sp*=specificity; OR=Odds Ratio

When fitting the Bayesian models, we used independent normal distributions for the logit sensitivity and the logit specificity. We set the mean of these distributions to be equal to the inverse logit of the parameters used for the scenarios. The standard deviation was set to 0.2 for the logit sensitivity. For example in the last scenario (16) this corresponds to $logit(Se_{V_{+}}) \sim N(3.66, 0.2)$, where $3.66=logit(0.975)$. The standard deviation for the logit specificity is 0.1 when specificity is equal to 0.99. The performance of the Bayesian models accounting for imperfect sensitivity and specificity was evaluated with respect to bias and coverage probability. Bias is expressed as the ratio between the estimated and the true OR (e.g., a bias of 1.5 means that the estimated OR is 50% higher than the true OR).

### Results

**Fig. 1:**
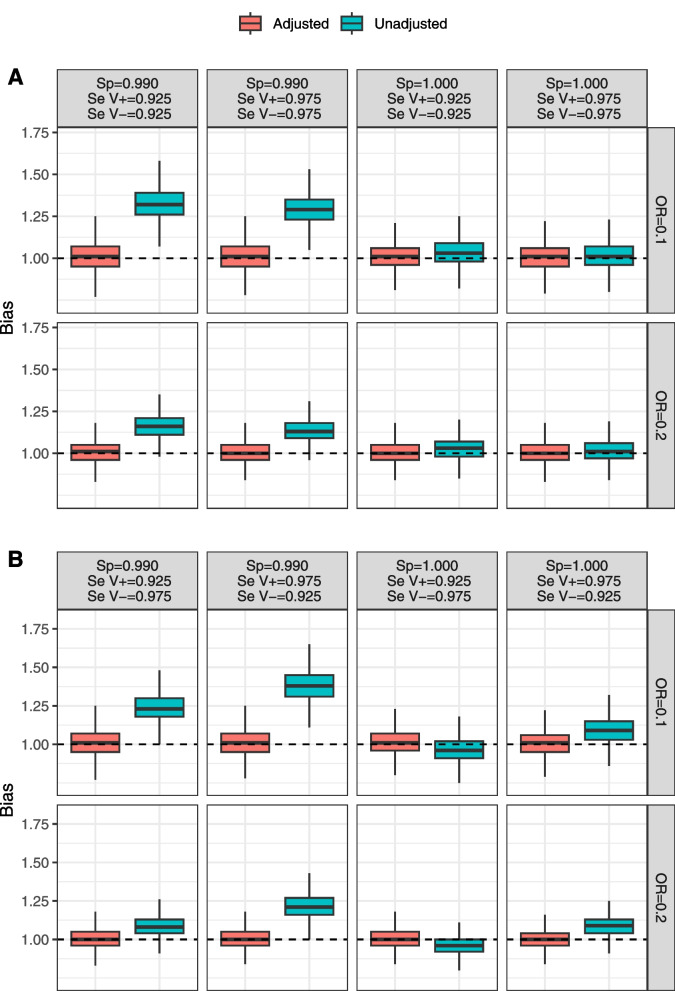
Results of simulations for *non-differential* (**A**) and *differential* (**B**) misclassification scenarios. Bias is defined as the ratio between the estimated and the true OR. Dashed horizontal indicates no bias. Abbreviations: $Se_{V_{-}}$=sensitivity in unvaccinated; $Se_{V_{+}}$=sensitivity in vaccinated; *Sp*=specificity; OR=Odds Ratio

Results of the simulations show that the degree of bias in estimating the effect of vaccination is zero or negligible for scenarios with perfect specificity (1.00) combined with non-differential misclassification bias resulting from imperfect sensitivity (Fig. 1A). In these scenarios, the median amount of bias ranged from 1.01 to 1.03.

However, in the presence of differential misclassification, the bias in vaccine effect is substantial, with a direction that depends on the combination of sensitivity values between vaccinated and unvaccinated subgroups (Fig. 1B). For example, when $Se_{V_{+}}=0.975$ and $Se_{V_{-}}=0.925$ the median bias is 1.09, while when $Se_{V_{+}}=0.925$ and $Se_{V_{-}}=0.975$ the median bias is 0.96.

For scenarios with specificity equal to 0.99, we always have a noticeable bias towards the null (Fig. 1), with the median bias in the scenarios ranging from 1.08 ($Se_{V_{+}}=0.925$, $Se_{V_{-}}=0.975$, true OR=0.2) to 1.38 ($Se_{V_{+}}=0.975$, $Se_{V_{-}}=0.925$, true OR=0.1).

Full details of the results of the simulation study are available from the Additional file 1.

## Illustrative example

A previous TND study conducted in Ontario, Canada, investigated the effectiveness of mRNA vaccines for Covid-19 (bnt162b2 and mrna-1273) against symptomatic SARS-CoV-2 infection [19]. The study combined provincial SARS-CoV-2 laboratory testing data, Covid-19 vaccination, and health administrative datasets. All Ontarians aged at least 16 years, eligible for provincial health insurance, not living in long-term care, and tested for SARS-CoV-2 between 14 December 2020 and 19 April 2021 were included. Those who tested positive for SARS-CoV-2 before 14 December 2020 were excluded from the analysis along with recipients of the ChAdOx1 vaccine. In addition, the analysis was restricted to individuals with at least one relevant Covid-19 symptom (based on self-report or observation, such as measured temperature) at the time of testing.

Of the test-positive subjects (cases), 57 were vaccinated and 51 220 were unvaccinated. In subjects testing negative (negative-controls), 3 817 were vaccinated and 251 541 were unvaccinated (Table 2). The estimated unadjusted OR was 0.07 (95% CI=0.06-0.09), which translates to a VE of 93% (95% CI=91-94). Adjusted analysis was performed with a multivariable regression logistic model, including covariates selected a-priori based on their presumed associations with SARS-CoV-2 infection and receipt of a Covid-19 vaccine. The adjusted VE estimate was 91% (95% CI=89-93).

**Table 2:** Cross-classification of subjects between vaccination status (2 doses of Covid-19 mRNA vaccines) and symptomatic SARS-CoV-2 infection (at least 7 days from the second dose)

	${\boldsymbol T}_{\boldsymbol{-}}$	${\boldsymbol T}_{\boldsymbol{+}}$
${\boldsymbol V}_{\boldsymbol{-}}$	251 541	51 220
${\boldsymbol V}_{\boldsymbol{+}}$	3 817	57

$V_{-}$=unvaccinated; $V_{+}$=vaccinated; $T_{+}$=positive results With SARS-CoV-2 RT-PCR test; $T_{-}$=negative results With SARS-CoV-2 RT-PCR test

In order to use our Bayesian model to analyze this dataset, some prior information is required for the diagnostic test characteristics. Kostoulas and colleagues [6] used a Bayesian latent class model to estimate the diagnostic accuracy of RT-PCR and lateral flow immunoassay tests for Covid-19. They estimated the sensitivity of RT-PCR to be 0.68 (95% PrI=0.63-0.73) and the specificity to be 0.99 (95% PrI=0.98-1.00). We plugged this prior information into our model by using a *Beta*(226.16, 105.93) prior for the sensitivity and a *Beta*(287.48, 2.14) prior for the specificity. Our estimated OR was 0.027 (95% PrI=0.000-0.054), which translates into a VE of 97.3% (95% PrI=94.6-100.0).

Alternative sources of prior information are also available. A recent analysis of Danish registry data used a Bayesian latent class model to estimate the diagnostic accuracy of RT-PCR and antigen tests for Covid-19 as used in the Danish Covid-19 control program [7]. Here, the specificity of RT-PCR was estimated to be close to 1.00, and the sensitivity estimates were 0.957 (95% PrI=0.928-0.984). We plugged this prior information into our model by using a *Beta*(3040.61, 3.64) prior for the specificity and a *Beta*(168.66, 6.84) prior for the sensitivity. This changed the estimated OR to 0.064 (95% PrI=0.035-0.090), which translates into a VE of 93.6% (95% PrI=91.0-96.5).

**Fig. 2:**
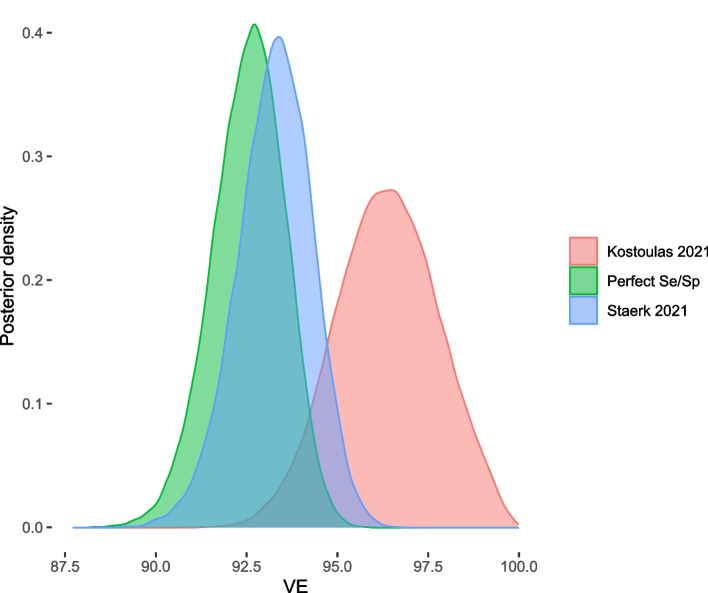
Posterior density of VE depending on the used priors for RT-PCR specificity and sensitivity. Abbreviations: VE=vaccine effectiveness

For all the models, a $Beta \sim (2,2)$ prior was used for the positive testing rate in vaccinated and unvaccinated individuals. The MCMC sampling algorithm required 100 000 iterations with burnin 50 000 and thin interval 25, which reduced autocorrelation to an acceptable level and provided a sufficient effective sample size. Assumptions and estimates of fitted models are reported in Table 3, while posterior distributions of VE are displayed in Fig. 2. Code and MCMC output is given in the Additional file 1.

**Table 3:** Results of the Bayesian model using different priors for RT-PCR specificity and sensitivity for addressing misclassification bias

Study	Sp/Se	Median (95% PrI)	OR (95% PrI)	VE (95% PrI)
Perfect classification	*Sp*	1.000	0.075 (0.057-0.096)	92.5 (90.4-94.3)
	*Se*	1.000		
Kostoulas 2021	*Sp*	0.990 (0.980-1.000)	0.036 (0.009-0.063)	96.4 (93.7-99.1)
	*Se*	0.680 (0.630-0.730)		
Stærk-Østergaard 2021	*Sp*	0.999 (0.999-1.000)	0.070 (0.050-0.090)	93.0 (91.0-95.0)
	*Se*	0.957 (0.928-0.984)		

*Se*=sensitivity; *Sp*=specificity; OR=Odds Ratio; PrI=probability interval; VE=vaccine effectiveness

## Discussion

We propose a statistical method for addressing potential misclassification bias in TND studies, which can be implemented in a Bayesian inference framework. Our example is motivated by the extensive application of TND studies to evaluate the effectiveness of Covid-19 vaccines, but we note that the method is equally applicable to TND studies for evaluating the effectiveness of any intervention for any disease, and can be adapted for use with other types of intervention study where the diagnostic test may be imperfect.

Although TND has been proven to be a valid alternative to cohort and case-control studies [8], several sources of bias can affect the results. One potential source of bias is confounding due to covariates related to vaccination and the outcome of interest [20]. Additionally, biased estimates could result from the fact that vaccination may modify the probability of seeking medical care against the disease because vaccinated patients may have less severe symptoms than unvaccinated patients [20]. In cases where tests with high analytical sensitivity are used to detect the condition of interest, individuals testing positive may be counted as cases even if their symptoms are not due to the virus [21].

The TND method is vulnerable to misclassification bias, and can therefore be expected to produce biased estimates of vaccine efficacy where any such factors exist. When the diagnostic tests used in the study are imperfect (i.e., have a sensitivity or a specificity less than 1), the most likely result of the resultant bias is an underestimation of VE [9]. Traditionally, diagnostic test sensitivity and specificity have been considered as being intrinsic to the diagnostic test, i.e., constant and universally applicable [22]. For example, PCR tests tend to have higher sensitivity than rapid antigen tests [5]. However, several additional factors are associated with overall field sensitivity and specificity of tests used for diagnosing Covid-19, which may not be due to the laboratory methods themselves. For example, proper collection and handling of samples are crucial for accurate test results [23], and using different PCR primers or antibody assays can affect test performance [5]. The sensitivity and specificity of a test can also be affected by the patient’s characteristics, such as age, comorbidities, and viral load [5, 24]. Crucially, many research designs such as cohort, case-control, randomized controlled trials, and TND, may be differentially impacted by the misclassification bias that this creates [9]. Due to these issues, the robustness of Covid-19 VE estimates from TND studies has previously been challenged [2, 4]. Therefore, a statistical method capable of dealing with these potential sources of bias significantly improves the current state of the art.

Our simulations and examples focused on the potential bias of OR estimates because VE is typically defined as a function of OR in TND studies. Risk difference and relative risk could also be biased along the same principles. However, it should also be noted that the relative risk is unbiased by definition any misclassification is non-differential in nature. Our simulations show that bias in VE estimates is unlikely if specificity is equal to 1 and there is non-differential misclassification in sensitivity. A small amount of bias can happen if specificity is equal to 1 and there is differential misclassification in sensitivity. If the specificity is equal to 0.99, bias is likely and non-negligible.

The results of the illustrative example show that the effectiveness of Covid-19 vaccines could be slightly or moderately underestimated, depending on the evidence used to inform the prior distributions of sensitivity and specificity in the model. In the example, we used two different sources of evidence for calibrating our prior distributions for test sensitivity and specificity. The estimates from the study of Kostoulas and colleagues [6] were based on a pooled analysis of diagnostic studies in the early phase of the pandemic. The pooled data were from retrospective case-control studies where cases were drawn from symptomatic patients admitted to the hospital. The estimates from Stærk-Østergaard and colleagues [7] were from data from the Danish COVID-19 surveillance system. The surveillance data were collected between February and June 2021, a period in which the Alpha variant dominated the pandemic in Denmark. Based on our results of different realistic scenarios of sensitivity and specificity of RT-PCR, we conclude that the published estimates of VE in TND Covid-19 studies should be relatively robust to misclassification bias. This is mainly due to the high specificity of RT-PCR. However, a slight deviation from perfect specificity could have a non-negligible impact on the results.

Although the proposed approach is easy to implement, it is worth noting that if imperfect test accuracy is taken into account, incorrect prior information can lead to unreliable posterior estimates [25]. Therefore, the prior distributions of sensitivity and specificity need to be carefully specified according to the best available evidence. A reasonable option may be to report results under different test sensitivity and specificity scenarios to ensure that the VE estimates are robust to any potential inaccuracies in specification of priors for sensitivity and specificity.

Several TND studies make use of multivariable logistic regression for obtaining adjusted OR. Our framework can easily accommodate this extension by modeling the dependency of the logit of the probability of testing positive on vaccination status and potential confounders. Another possible extension is the implementation in prospective cohorts or phase 3 trials. For example, a Bayesian beta-binomial approach was adopted in a phase 3 trial to evaluate the safety end efficacy of the BNT162b2 mRNA Covid-19 vaccine [26]. Our approach can be easily implemented also in such a situation by fitting a Poisson regression and considering the incidence rate ratio instead of OR as a measure of effectiveness.

In conclusion, we suggest that similar approaches to the one proposed here should be adopted in future TND studies in order to reduce the potential impact of differential misclassification bias on estimates of VE. Such studies should also incorporate sensitivity analyses to ensure that conclusions are robust to the choice of assumptions in the analysis.

## Supplementary Information

**Additional file 1.** R code for reproducing the analysis of example data and detailed results of the simulation study.

## Data Availability

The R code needed to replicate the study is publicly available via a GitHub repository https://github.com/paoloeusebi/tnd-vaccine-effectiveness.

## Abbreviations

$$n_{V_{-}}$$: Unvaccinated
$$n_{V_{+}}$$: Vaccinated
$$\text{OR}$$: Odds ratio
$p_{V_{-}}$: True probability of disease in unvaccinated individuals
$p_{V_{+}}$: True probability of disease in vaccinated individuals
$$\text{PrI}$$: Probability interval
$\text{RT-PCR}$: Reverse transcriptase-polymerase chain reaction
$\text{SARS-CoV-2}$: Severe acute respiratory syndrome coronavirus
$$Se$$: Sensitivity
$Se_{V_{-}}$: Sensitivity in unvaccinated individuals
$Se_{V_{+}}$: Sensitivity in vaccinated individuals
$$Sp$$: Specificity
$Sp_{V_{-}}$: Specificity in unvaccinated individuals
$Sp_{V_{+}}$: Specificity in vaccinated individuals
$\text{TND}$: Test-negative design
$$\text{VE}$$: Vaccine effectiveness
$y_{V_{-}}$: Positive tests in unvaccinated individuals
$y_{V_{+}}$: Positive tests in vaccinated individuals

## References

[1] Chua H, Feng S, Lewnard JA, Sullivan SG, Blyth CC, Lipsitch M (2020). The use of test-negative controls to monitor vaccine effectiveness: a systematic review of methodology. Epidemiology (Cambridge, Mass)..

[2] Patel MM, Jackson ML, Ferdinands J (2020). Postlicensure Evaluation of COVID-19 Vaccines. JAMA..

[3] Sullivan SG, Feng S, Cowling BJ (2014). Potential of the test-negative design for measuring influenza vaccine effectiveness: a systematic review. Expert Rev Vaccines..

[4] Dean NE, Hogan JW, Schnitzer ME (2021). Covid-19 vaccine effectiveness and the test-negative design. N Engl J Med..

[5] Kucirka LM, Lauer SA, Laeyendecker O, Boon D, Lessler J (2020). Variation in false-negative rate of reverse transcriptase polymerase chain reaction-based SARS-CoV-2 tests by time since exposure. Ann Intern Med..

[6] Kostoulas P, Eusebi P, Hartnack S (2021). Diagnostic Accuracy Estimates for COVID-19 Real-Time Polymerase Chain Reaction and Lateral Flow Immunoassay Tests With Bayesian Latent-Class Models. Am J Epidemiol..

[7] Stærk-Østergaard J, Kirkeby C, Engbo Christiansen L, Asger Andersen M, Holten Møller C, Voldstedlund M, et al. Evaluation of diagnostic test procedures for SARS-CoV-2 using latent class models. J Med Virol. 2022;94(10):4754–61. 10.1002/jmv.27943 PMC9349895 35713189

[8] Jackson ML, Nelson JC (2013). The test-negative design for estimating influenza vaccine effectiveness. Vaccine..

[9] Lewnard JA, Patel MM, Jewell NP, Verani JR, Kobayashi M, Tenforde MW (2021). Theoretical framework for retrospective studies of the effectiveness of SARS-CoV-2 vaccines. Epidemiology (Cambridge, Mass)..

[10] Choy SL, O’Leary R, Mengersen K (2009). Elicitation by design in ecology: using expert opinion to inform priors for Bayesian statistical models. Ecology..

[11] Garthwaite PH, Kadane JB, O’Hagan A (2005). Statistical methods for eliciting probability distributions. J Am Stat Assoc..

[12] Kuhnert PM, Martin TG, Griffiths SP (2010). A guide to eliciting and using expert knowledge in Bayesian ecological models. Ecol Lett..

[13] Gelman A, Carpenter B (2020). Bayesian analysis of tests with unknown specificity and sensitivity. J R Stat Soc Ser C (Appl Stat)..

[14] Kostoulas P, Nielsen SS, Branscum AJ, Johnson WO, Dendukuri N, Dhand NK (2017). STARD-BLCM: Standards for the Reporting of Diagnostic accuracy studies that use Bayesian Latent Class Models. Prev Vet Med..

[15] Plummer M, et al. JAGS: A program for analysis of Bayesian graphical models using Gibbs sampling. In: Proceedings of the 3rd international workshop on distributed statistical computing. vol. 124. Vienna; 2003. p. 1–10.

[16] R Core Team. R: A Language and Environment for Statistical Computing. Vienna; 2021. https://www.R-project.org/.

[17] Denwood MJ (2016). runjags: An R package providing interface utilities, model templates, parallel computing methods and additional distributions for MCMC models in JAGS. J Stat Softw..

[18] Kostoulas P. PriorGen: Generates Prior Distributions for Proportions. R package version 1.1.2. 2018. https://CRAN.R-project.org/package=PriorGen.

[19] Chung H, He S, Nasreen S, Sundaram ME, Buchan SA, Wilson SE, et al. Effectiveness of BNT162b2 and mRNA-1273 covid-19 vaccines against symptomatic SARS-CoV-2 infection and severe covid-19 outcomes in Ontario, Canada: test negative design study. BMJ. 2021;374. 10.1136/bmj.n1943. 10.1136/bmj.n1943 PMC8377789 34417165

[20] Orenstein EW, De Serres G, Haber MJ, Shay DK, Bridges CB, Gargiullo P (2007). Methodologic issues regarding the use of three observational study designs to assess influenza vaccine effectiveness. Int J Epidemiol..

[21] Ainslie KE, Shi M, Haber M, Orenstein WA (2017). On the bias of estimates of influenza vaccine effectiveness from test-negative studies. Vaccine..

[22] Berkvens D, Speybroeck N, Praet N, Adel A, Lesaffre E. Estimating disease prevalence in a Bayesian framework using probabilistic constraints. Epidemiology. 2006;17(2):145–53. 10.1097/01.ede.0000198422.64801.8d. 10.1097/01.ede.0000198422.64801.8d 16477254

[23] Lippi G, Simundic AM, Plebani M (2020). Potential preanalytical and analytical vulnerabilities in the laboratory diagnosis of coronavirus disease 2019 (COVID-19). Clin Chem Lab Med (CCLM)..

[24] Irwin N, Murray L, Ozynski B, Richards GA, Paget G, Venturas J, et al. Age significantly influences the sensitivity of SARS-CoV-2 rapid antibody assays. Int J Infect Dis. 2021;109:304–9. 10.1016/j.ijid.2021.07.027 PMC8276555 34271199

[25] Speybroeck N, Devleesschauwer B, Joseph L, Berkvens D (2013). Misclassification errors in prevalence estimation: Bayesian handling with care. Int J Public Health..

[26] Polack FP, Thomas SJ, Kitchin N, Absalon J, Gurtman A, Lockhart S (2020). Safety and efficacy of the BNT162b2 mRNA Covid-19 vaccine. N Engl J Med..

