# Supplementary material for: Addressing misclassification bias in vaccine effectiveness studies with an application to Covid-19
Source: BMC Med Res Methodol. 2023 Feb 27;23:55. doi: 10.1186/s12874-023-01853-4 (PMC9969950; doi:10.1186/s12874-023-01853-4)
Supplement: Supplementary file 1 — Additional file 1. R code for reproducing the analysis of example data and detailed results of the simulation study. [file 12874_2023_1853_MOESM1_ESM.pdf]

Supplementary Information

Addressing Misclassification Bias in Vaccine Effectiveness Studies with an application to Covid-19

Paolo Eusebi, Niko Speybroeck, Matthew Denwood, Jacob Stærk-Østergaard, Sonja Hartnack, Polychronis Kostoulas

Corresponding author: Paolo Eusebi, University of Perugia, Italy paoloeusebi@gmail.com

Load packages/functions

```
library(tidyverse)
library(runjags)
library(rjags)
library(rootSolve)
library(parallel)
library(kableExtra)
source("R/functions.R")
testjags()
```

```
## You are using R version 4.2.2 (2022-10-31 ucrt) on a windows machine,
## with the RTerm GUI
## JAGS version 4.3.1 found successfully using the command 'C:/Program
## Files/JAGS/JAGS-4.3.1/x64/bin/jags-terminal.exe'
## The rjags package is installed
```

Results from simulation study

Table 1: Results from simulation studies

	Sp	Se V+	Se V-	True OR	Bias - BM Adjusted	Bias - Unadjusted
Non-differential misclassification	0.99	0.925	0.925	0.1	1.01 (0.82-1.20)	1.32 (1.12-1.53)
	0.99	0.925	0.925	0.2	1.01 (0.87-1.17)	1.16 (1.02-1.32)
	0.99	0.975	0.975	0.1	1.01 (0.83-1.20)	1.29 (1.10-1.49)
	0.99	0.975	0.975	0.2	1.00 (0.87-1.15)	1.13 (1.00-1.28)
	1.00	0.925	0.925	0.1	1.01 (0.85-1.18)	1.03 (0.87-1.21)
	1.00	0.925	0.925	0.2	1.00 (0.89-1.15)	1.03 (0.91-1.17)
	1.00	0.975	0.975	0.1	1.01 (0.85-1.18)	1.01 (0.86-1.18)
	1.00	0.975	0.975	0.2	1.00 (0.89-1.15)	1.01 (0.89-1.15)
Differential missclassification	0.99	0.925	0.975	0.1	1.01 (0.83-1.20)	1.23 (1.05-1.43)
	0.99	0.925	0.975	0.2	1.00 (0.88-1.16)	1.08 (0.96-1.23)
	0.99	0.975	0.925	0.1	1.01 (0.82-1.21)	1.38 (1.17-1.59)
	0.99	0.975	0.925	0.2	1.00 (0.87-1.16)	1.21 (1.06-1.38)
	1.00	0.925	0.975	0.1	1.01 (0.86-1.19)	0.96 (0.82-1.13)
	1.00	0.925	0.975	0.2	1.00 (0.89-1.15)	0.96 (0.85-1.09)
	1.00	0.975	0.925	0.1	1.01 (0.85-1.17)	1.09 (0.92-1.26)
	1.00	0.975	0.925	0.2	1.00 (0.89-1.14)	1.09 (0.96-1.23)

BM = Bayesian model; Se V− = sensitivity in unvaccinated; Se V+ = sensitivity in vaccinated; Sp = specificity; OR = Odds Ratio
Bias statistics are median (5th - 95th percentiles) of the 1000 simulations ran for each scenario

Code and MCMC output of illlustrative case

Gibbs sampling set-up.

The MCMC sampling required 100 000 iterations with 50 000 burnin and thin interval 25.

```
inits1 <- list(".RNG.name" = "base::Mersenne-Twister", ".RNG.seed" = 100022)
inits2 <- list(".RNG.name" = "base::Mersenne-Twister", ".RNG.seed" = 300022)

n_thin <- 25
n_burnin <- 50000
n_samples <- 100000
```

Data from Chung et al. (2021).

A TND study conducted in Ontario, Canada, investigated the effectiveness of mRNA Covid-19 vaccines (bnt162b2 and mrna-1273) against symptomatic SARS-CoV-2 infection. (Chung et al. 2021) The study used linked data from provincial SARS-CoV-2 laboratory testing, Covid-19 vaccination, and health administrative datasets.

In testing positve subjects (cases) 57 were vaccinated and 51 220 unvaccinated. In subjects testing negative (negative-controls) 3 817 were vaccinated and 251 541 unvaccinated

```
y <- matrix(
  c(51220, 251541, 57, 3817),
  nrow = 2,
  byrow = T,
  dimnames = list(c("V-", "V+"), c("T+", "T-"))
)
y
```

```
##        T+     T-
## V- 51220 251541
## V+    57   3817
```

```
N <- apply(y, 1, sum)
N
```

```
##     V-     V+
## 302761   3874
```

Bayesian models written in JAGS

```
# Bayesian model assuming perfect classification --------------------------

bm_1t_perf <- " model {

    for (i in 1:2) {

  # likelihood
    y[i,1] ~ dbin(pi[i], N[i])

  # priors for prevalence parameters
    pi[i] ~ dbeta(2,2)
  }

  # Computing OR/VE

    OR <- (pi[2]/(1-pi[2])) / (pi[1]/(1-pi[1]))
    VE <- (1-OR)*100

  #data# N, y
  #inits#
  #monitor# pi, OR, VE

  }
"
```

```
# Bayesian model for non-differential misclassification -------------------

bm_1t_nondif <- " model {

    for (i in 1:2) {

  # likelihood
    y[i,1] ~ dbin(prob[i], N[i])
    prob[i] <- pi[i]*Se + (1-pi[i])*(1-Sp)

  # priors for prevalence parameters
    pi[i] ~ dbeta(2,2)
  }

  # priors for Se and Sp
    Se~dbeta(HPSe[1], HPSe[2])
    Sp~dbeta(HPSp[1], HPSp[2])

  # Computing OR/VE

    OR <- (pi[2]/(1-pi[2])) / (pi[1]/(1-pi[1]))
    VE <- (1-OR)*100

  #data# N, y, HPSe, HPSp
  #inits#
  #monitor# Se, Sp, pi, OR, VE

  }
"
```

Model 1: perfect classification

```
res_perfect <- run.jags(
  bm_1t_perf,
  n.chains = 2,
  inits = list(inits1, inits2),
  burnin = n_burnin,
  sample = n_samples,
  thin = n_thin
)
```

```
## Compiling rjags model...
## Calling the simulation using the rjags method...
## Adapting the model for 1000 iterations...
## Burning in the model for 50000 iterations...
## Running the model for 2500000 iterations...
## Simulation complete
## Calculating summary statistics...
## Calculating the Gelman-Rubin statistic for 4 variables....
## Finished running the simulation
```

```
round(summary(res_perfect), 3)
```

```
##       Lower95 Median Upper95   Mean    SD Mode MCerr MC%ofSD SSeff AC.250 psrf
## pi[1]   0.168  0.169   0.171  0.169 0.001   NA 0.000     0.7 20000  0.002    1
## pi[2]   0.012  0.015   0.019  0.015 0.002   NA 0.000     0.7 20405  0.002    1
## OR      0.057  0.075   0.096  0.076 0.010   NA 0.000     0.7 20382  0.002    1
## VE     90.409 92.460  94.269 92.404 0.996   NA 0.007     0.7 20382  0.002    1
```

```
plot(
  res_perfect,
  plot.type = c("histogram","trace", "autocorr"),
  vars = c("pi", "OR", "VE"),
  layout = c(4, 3)
)
```

```
## Generating plots...
```

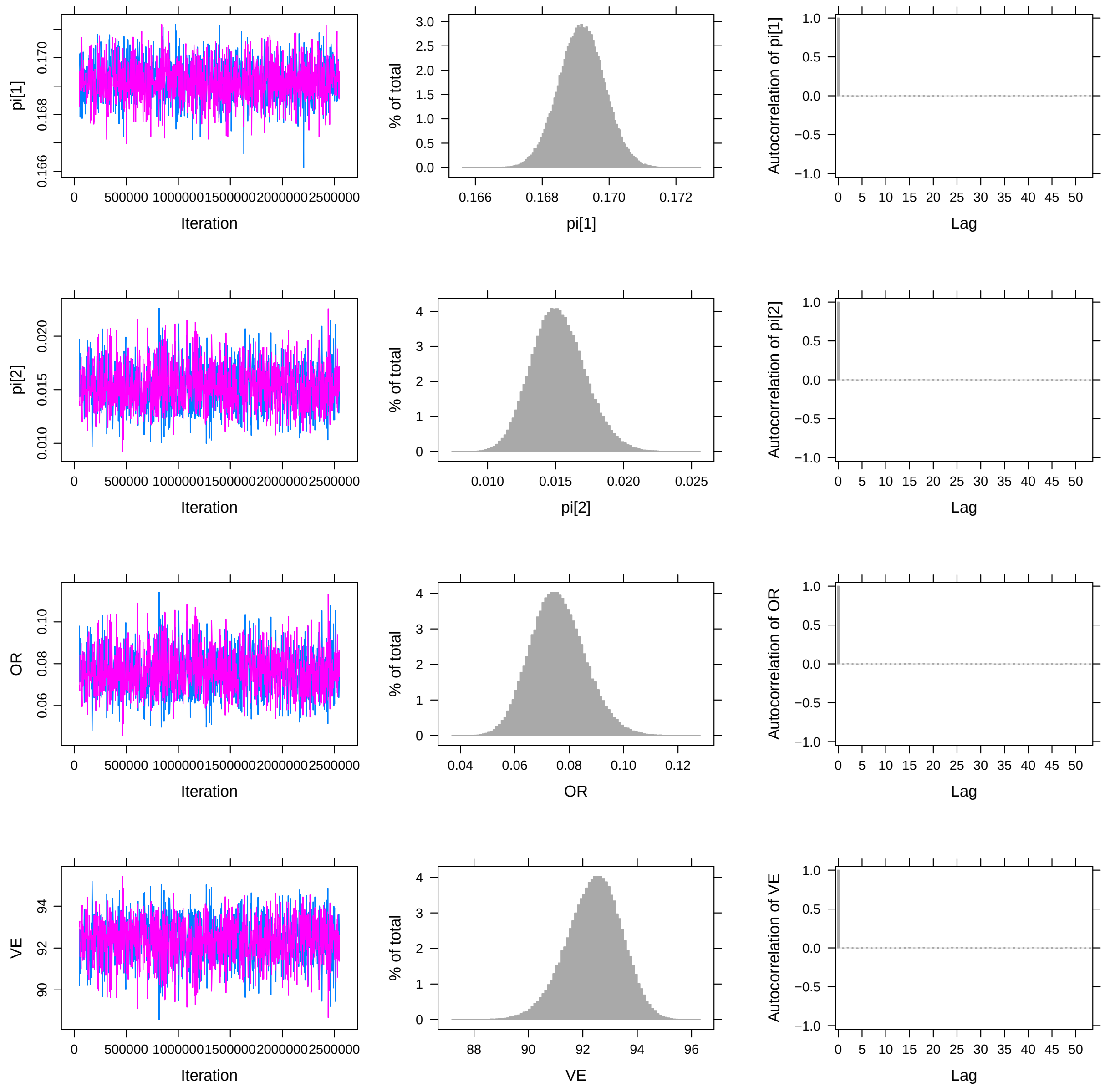

pi[1]
Iteration
% of total
Autocorrelation of pi[1]
Lag
pi[2]
Autocorrelation of pi[2]
OR
Autocorrelation of OR
VE
Autocorrelation of VE

Model 2: sensitivity and specificity from Kostoulas, Eusebi, and Hartnack (2021)

Kostoulas and colleagues (Kostoulas, Eusebi, and Hartnack 2021) used a Bayesian latent class model to estimate the diagnostic accuracy of RT-PCR and lateral flow immunoassay tests for Covid-19. The sensitivity of RT-PCR was 0.68 (95% PrI=0.63-0.73), while the specificity was 0.99 (95% PrI=0.98-1.00).

We plugged-in this prior information in our model by using a Beta(226.16, 105.93) prior for the sensitivity and a Beta(287.48, 2.14) prior for the specificity.

```
HPSe <- findbetaqq2(
  percentile.value1 = 0.63,
  percentile1 = 0.025,
  percentile.value2 = 0.73,
  percentile2 = 0.975
)
HPSe
```

```
## [1] 226.16 105.93
```

```
round(qbeta(c(0.025, 0.5, 0.975), # check
            HPSe[1], HPSe[2]), 2)
```

```
## [1] 0.63 0.68 0.73
```

```
HPSp <- findbeta2(
    themedian = 0.99,
    percentile = 0.975,
    lower.v = FALSE,
    percentile.value = 0.98
  )
HPSp
```

```
## [1] 606.34   6.45
```

```
round(qbeta(c(0.025, 0.5, 0.975), # check
            HPSp[1], HPSp[2]), 3)
```

```
## [1] 0.980 0.990 0.996
```

```
res_kostoulas <- run.jags(
  bm_1t_nondif,
  n.chains = 2,
  inits = list(inits1, inits2),
  burnin = n_burnin,
  sample = n_samples,
  thin = n_thin
)
```

```
## Compiling rjags model...
## Calling the simulation using the rjags method...
## Adapting the model for 1000 iterations...
```

```
## Burning in the model for 50000 iterations...
## Running the model for 2500000 iterations...
## Simulation complete
## Calculating summary statistics...
## Calculating the Gelman-Rubin statistic for 6 variables....
## Note: Unable to calculate the multivariate psrf
## Finished running the simulation
```

```
round(summary(res_kostoulas), 3)
```

```
##       Lower95 Median Upper95   Mean    SD Mode MCerr MC%ofSD SSeff AC.250 psrf
## Se      0.626  0.678   0.728  0.677 0.026   NA  0.00     0.8 14469  0.160    1
## Sp      0.987  0.992   0.997  0.992 0.003   NA  0.00     0.7 20000  0.004    1
## pi[1]   0.223  0.241   0.262  0.241 0.010   NA  0.00     0.8 14725  0.167    1
## pi[2]   0.002  0.011   0.020  0.011 0.004   NA  0.00     0.7 20000  0.012    1
## OR      0.009  0.036   0.063  0.036 0.014   NA  0.00     0.7 20000  0.004    1
## VE     93.700 96.405  99.051 96.384 1.391   NA  0.01     0.7 20000  0.004    1
```

```
plot(
  res_kostoulas,
  plot.type = c("histogram","trace", "autocorr"),
  vars = c("Se", "Sp", "pi", "OR", "VE"),
  layout = c(6, 3)
)
```

```
## Generating plots...
```

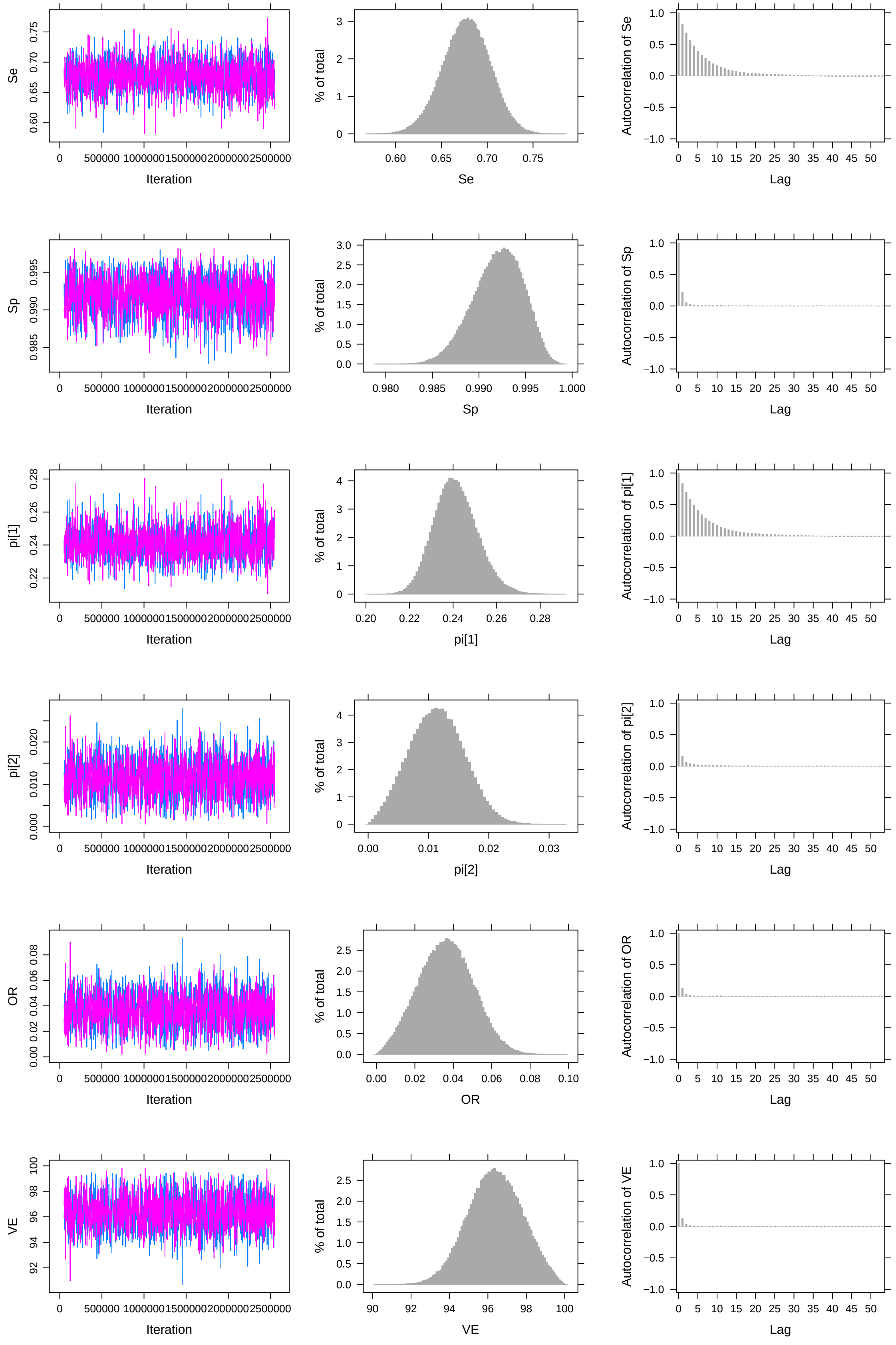

Se
Iteration
% of total
Autocorrelation of Se
Lag
Sp
Autocorrelation of Sp
pi[1]
Autocorrelation of pi[1]
pi[2]
Autocorrelation of pi[2]
OR
Autocorrelation of OR
VE
Autocorrelation of VE

Model 3: sensitivity and specificity from Stærk-Østergaard et al. (2021)

A recent report using Danish registries data used a Bayesian latent class model to estimate the diagnostic accuracy of RT-PCR and antigen tests for Covid-19.(Stærk-Østergaard et al. 2021) The specificity of RT-PCR was estimated to be close to 1.00. The sensitivity estimates were 0.957 (95% PrI=0.928-0.984). We plugged-in this prior information in our model by using a Beta(3040.61, 3.64) prior for the specificity and a Beta(168.66, 6.84) prior for the sensitivity.

```
# Sp
HPSp <- findbetaqq2(
  percentile.value1 = 0.9973,
  percentile1 = 0.025,
  percentile.value2 = 0.9997,
  percentile2 = 0.975
)
HPSp
```

```
## [1] 3040.61    3.64
```

```
round(qbeta(c(0.025, 0.5, 0.975),
            HPSp[1], HPSp[2]), 4)
```

```
## [1] 0.9973 0.9989 0.9997
```

```
# Se
HPSe <- findbetaqq2(
  percentile.value1 = 0.9279,
  percentile1 = 0.025,
  percentile.value2 = 0.9843,
  percentile2 = 0.975
)
HPSe
```

```
## [1] 168.66   6.84
```

```
round(qbeta(c(0.025, 0.5, 0.975),
            HPSe[1], HPSe[2]), 4)
```

```
## [1] 0.9279 0.9628 0.9843
```

```
res_staerk <- run.jags(
  bm_1t_nondif,
  n.chains = 2,
  inits = list(inits1, inits2),
  burnin = n_burnin,
  sample = n_samples,
  thin = n_thin
)
```

```
## Compiling rjags model...
## Calling the simulation using the rjags method...
```

```
## Adapting the model for 1000 iterations...
## Burning in the model for 50000 iterations...
## Running the model for 2500000 iterations...
## Simulation complete
## Calculating summary statistics...
## Calculating the Gelman-Rubin statistic for 6 variables....
## Note: Unable to calculate the multivariate psrf
## Finished running the simulation
```

```
round(summary(res_staerk), 3)
```

```
##        Lower95 Median Upper95   Mean    SD Mode MCerr MC%ofSD SSeff AC.250 psrf
## Se       0.931  0.962   0.987  0.960 0.015   NA 0.000     0.7 20000  0.000    1
## Sp       0.998  0.999   1.000  0.999 0.001   NA 0.000     0.7 20598  0.000    1
## pi[1]    0.170  0.175   0.181  0.175 0.003   NA 0.000     0.7 20000  0.002    1
## pi[2]    0.011  0.015   0.019  0.015 0.002   NA 0.000     0.7 20000  0.000    1
## OR       0.050  0.070   0.090  0.070 0.010   NA 0.000     0.7 20000  0.000    1
## VE      90.965 93.030  95.006 92.993 1.033   NA 0.007     0.7 20000  0.000    1
```

```
plot(
  res_staerk,
  plot.type = c("histogram","trace", "autocorr"),
  vars = c("Se", "Sp", "pi", "OR", "VE"),
  layout = c(6, 3)
)
```

```
## Generating plots...
```

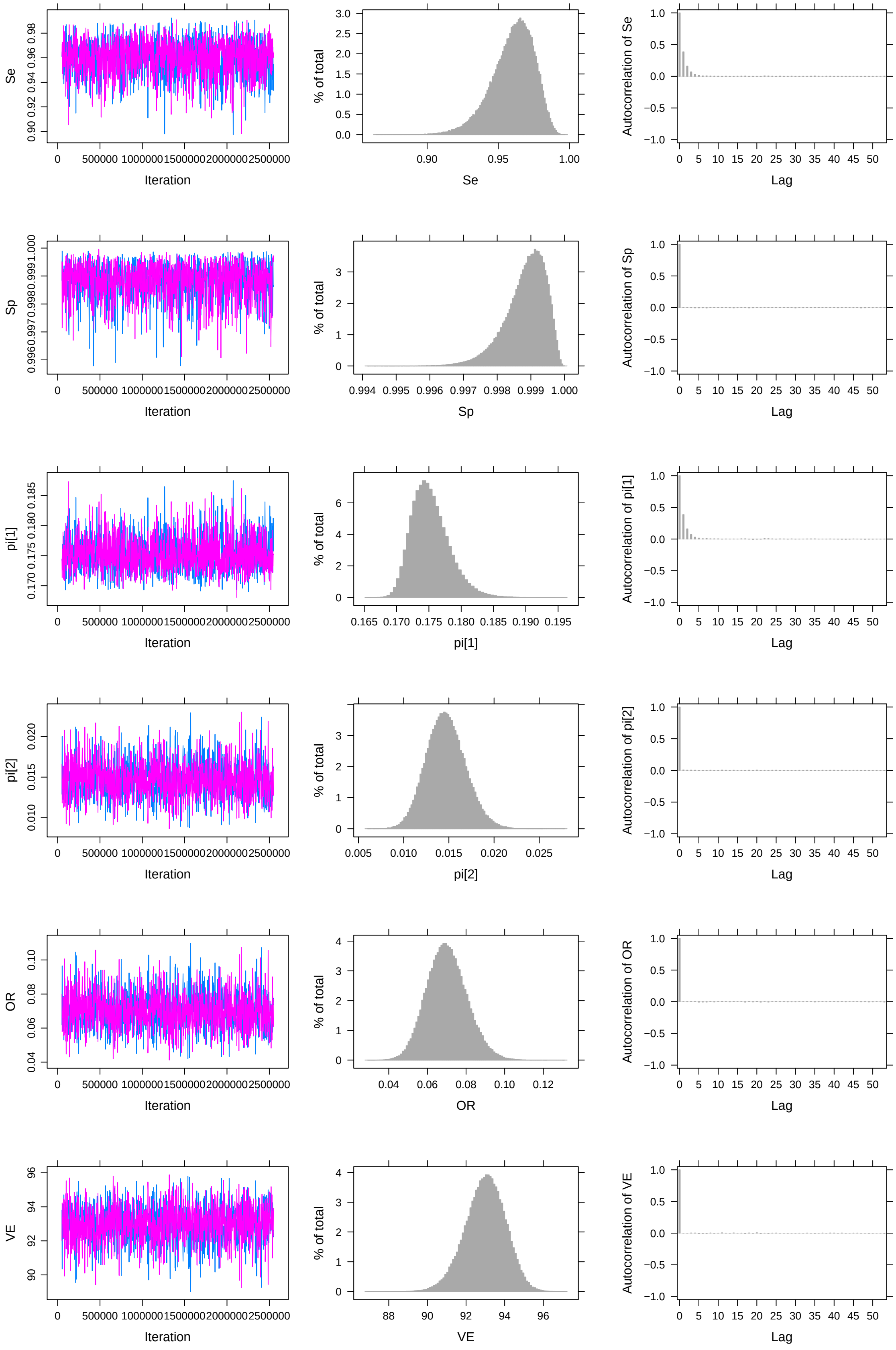
Se
Iteration
% of total
Autocorrelation of Se
Lag
Sp
Autocorrelation of Sp
pi[1]
Autocorrelation of pi[1]
pi[2]
Autocorrelation of pi[2]
OR
Autocorrelation of OR
VE
Autocorrelation of VE

Code availability

Code available at https://github.com/paoloeusebi/tnd-vaccine-effectiveness/

References

Chung, Hannah, Siyi He, Sharifa Nasreen, Maria E Sundaram, Sarah A Buchan, Sarah E Wilson, Branson Chen, et al. 2021. "Effectiveness of BNT162b2 and mRNA-1273 Covid-19 Vaccines Against Symptomatic SARS-CoV-2 Infection and Severe Covid-19 Outcomes in Ontario, Canada: Test Negative Design Study." *BMJ* 374. https://doi.org/10.1136/bmj.n1943.

Kostoulas, Polychronis, Paolo Eusebi, and Sonja Hartnack. 2021. "Diagnostic Accuracy Estimates for COVID-19 Real-Time Polymerase Chain Reaction and Lateral Flow Immunoassay Tests With Bayesian Latent-Class Models." *American Journal of Epidemiology* 190 (8): 1689–95. https://doi.org/10.1093/aje/kwab093.

Stærk-Østergaard, Jacob, Carsten Kirkeby, Lasse Engbo Christiansen, Michael Asger Andersen, Camilla Holten Møller, Marianne Voldstedlund, and Matthew J Denwood. 2021. "Evaluation of Diagnostic Test Procedures for SARS-CoV-2 Using Latent Class Models: Comparison of Antigen Test Kits and Sampling for PCR Testing Based on Danish National Data Registries." *arXiv Preprint arXiv:2112.11298*.
